# Quantum Dot Self‐Assembly Enables Low‐Threshold Lasing

**DOI:** 10.1002/advs.202101125

**Published:** 2021-08-27

**Authors:** Chun Zhou, Joao M. Pina, Tong Zhu, Darshan H. Parmar, Hao Chang, Jie Yu, Fanglong Yuan, Golam Bappi, Yi Hou, Xiaopeng Zheng, Jehad Abed, Hao Chen, Jian Zhang, Yuan Gao, Bin Chen, Ya‐Kun Wang, Haijie Chen, Tianju Zhang, Sjoerd Hoogland, Makhsud I. Saidaminov, Liaoxin Sun, Osman M. Bakr, Hongxing Dong, Long Zhang, Edward H. Sargent

**Affiliations:** ^1^ Key Laboratory of Materials for High‐Power Laser Shanghai Institute of Optics and Fine Mechanics Chinese Academy of Sciences Shanghai 201800 China; ^2^ The Edward S. Rogers Department of Electrical and Computer Engineering University of Toronto 10 King's College Road Toronto Ontario M5S 3G4 Canada; ^3^ University of Chinese Academy of Sciences No.19(A) Yuquan Road, Shijingshan District Beijing 100049 China; ^4^ Division of Physical Sciences and Engineering (PSE) King Abdullah University of Science and Technology (KAUST) Thuwal 23955‐6900 Kingdom of Saudi Arabia; ^5^ State Key Laboratory of Infrared Physics Shanghai Institute of Technical Physics Chinese Academy of Sciences Shanghai 200083 China; ^6^ Department of Chemistry and Electrical & Computer Engineering Centre for Advanced Materials and Related Technologies (CAMTEC) University of Victoria Victoria British Columbia V8P 5C2 Canada

**Keywords:** Auger recombination, lasing, perovskites, quantum dots, superlattices

## Abstract

Perovskite quantum dots (QDs) are of interest for solution‐processed lasers; however, their short Auger lifetime has limited lasing operation principally to the femtosecond temporal regime the photoexcitation levels to achieve optical gain threshold are up to two orders of magnitude higher in the nanosecond regime than in the femtosecond. Here the authors report QD superlattices in which the gain medium facilitates excitonic delocalization to decrease Auger recombination and in which the macroscopic dimensions of the structures provide the optical feedback required for lasing. The authors develope a self‐assembly strategy that relies on sodiumd—an assembly director that passivates the surface of the QDs and induces self‐assembly to form ordered three‐dimensional cubic structures. A density functional theory model that accounts for the attraction forces between QDs allows to explain self‐assembly and superlattice formation. Compared to conventional organic‐ligand‐passivated QDs, sodium enables higher attractive forces, ultimately leading to the formation of micron‐length scale structures and the optical faceting required for feedback. Simultaneously, the decreased inter‐dot distance enabled by the new ligand enhances exciton delocalization among QDs, as demonstrated by the dynamically red‐shifted photoluminescence. These structures function as the lasing cavity and the gain medium, enabling nanosecond‐sustained lasing with a threshold of 25 µJ cm^–2^.

## Graphical abstract

The manipulation of the surface properties of quantum dots can be used to control their self‐assembly into macroscopic structures that provide the required optical feedback for lasing. Here the use of a ligand that passivates the quantum dot surface and simultaneously induce self‐assembly into superlattice structures that sustain low‐threshold lasing is reported.

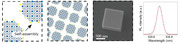

## 1 Introduction

Perovskite quantum dots (QDs) feature a low degeneracy of electronic states, near‐unity photoluminescence (PL) quantum yield (QY), and narrow emission linewidth, making them a promising material system for next‐generation lasers.^[^ ^1^, ^2^, ^3^, ^4^ ^]^ Since early reports of controlled synthesis,^[^ ^5^ ^]^ amplified spontaneous emission (ASE), and lasing^[^ ^1^ ^]^ in all‐inorganic cesium–lead halide quantum dots, further efforts have been devoted to achieving low lasing thresholds in longer‐lived excitation regimes.^[^ ^6^, ^7^ ^]^ Due to Auger recombination, however, the lasing threshold increases up to two orders of magnitude from short‐pulsed excitation (femtosecond) to long‐pulsed (nanosecond) excitation.^[^ ^6^ ^]^ Strategies such as shell growth and interface engineering suppress nonradiative recombination in metal chalcogenides and III–V nanocrystals,^[^ ^8^, ^9^, ^10^, ^11^, ^12^ ^]^ but the lack of lattice‐matched shell materials has prevented their application to perovskite quantum dots. As a result, lasing reports with perovskite QDs at or near continuous‐wave (CW) operation regimes remain a few.^[^ ^6^, ^13^, ^14^ ^]^ In contrast, room‐temperature CW lasing using thin‐film perovskites has been achieved.^[^ ^15^ ^]^

Self‐assembly of QDs into superlattice structures (SLs) leads to improved radiative properties and superfluorescence.^[^ ^16^, ^17^ ^]^ However, the large organic ligands passivating the surface of these QDs induce substantial spatial confinement, which in turn leads to high Auger recombination rates.^[^ ^18^ ^]^ The long‐chain organic ligands equally prevent heat dissipation, inducing large thermal loads that have acute effects when temporally long excitation pulses are used. Given that Auger recombination is highly thermally activated,^[^ ^19^ ^]^ the increased thermal load leads to a runaway increase in required input power to achieve population inversion. Additionally, the long ligands produce lower packing factors (volume of QDs per unit volume), reducing the overall refractive index of the laser active layer.^[^ ^20^ ^]^ This, in turn, reduces modal confinement, further increasing the threshold lasing fluence.

Substitution of the long‐chain organic ligands with inorganic compounds can increase the packing factor and reduce Auger recombination, helping to address these limitations.

Here we devise a ligand exchange strategy to replace the organic ligands passivating the surface of CsPbBr_3_ QDs with alkali metal cations (Na^+^). Sodium destabilizes the colloid and induces a process of QD self‐assembly to produce highly ordered, densely packed, 3D superlattices. Density functional theory (DFT) studying the thermodynamic origin of the self‐assembly process reveals that the presence of Na^+^ significantly increases the attraction between dots, promoting the formation of superstructures. Unlike superlattices of QDs passivated with organic ligands, the atomically passivated QDs form highly surface‐regular structures, which are suitable lasing microcavities. The superlattices show redshifted PL due to the delocalization of electrons and holes. The wavefunction delocalization, coupled with better thermal dissipation, leads to reduced Auger recombination. As we increase optical excitation power into the biexciton regime needed for gain, we find that the radiative QY of the superlattices remains high, while the QY of uncoupled QDs is diminished. Improved radiative properties obtained through exciton delocalization enable lasing at room temperature—something not achieved in previous superlattice lasing reports.^[^ ^17^ ^]^ Other superlattice formation methodologies based on organic ligands^[^ ^16^ ^]^ are equally unable to achieve room‐temperature lasing action. In contrast to perovskite thin films, perovskite QDs, with their abundant surfaces, can be engineered to control self‐assembly into macroscopic structures that provide the optical feedback required for lasing. The SLs also retain the advantages of QDs as gain materials, such as high oscillator strength and bandgap tunability via size control. Using these superlattices, we achieve room‐temperature nanosecond‐sustained lasing with a threshold of ≈25 µJ cm^–2^.

## 2 Results

### 2.1 Self‐Assembly of Quantum Dots

**Figure 1:**
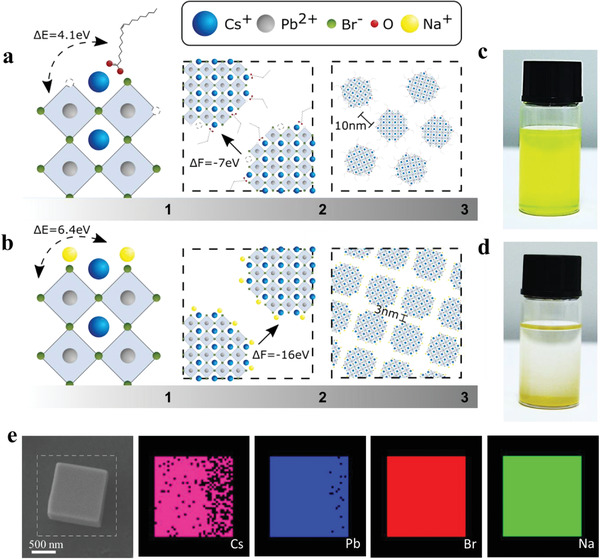
Effect of ligands on the self‐assembly interactions of quantum dots. a) Schematic illustration of 1) binding energy (Δ*E*) for surface passivation of QDs with oleic acid, 2) the interaction forces (Δ*F*) between neighboring QDs, and 3) distribution of QDs in films. b.1–b.3) Corresponding illustration of QDs passivated using sodium ligands. c) Original solution of quantum dots passivated with long‐chain organic ligands. d) Formation of precipitates in solution after ligand exchange. e) EDS reveals the presence of sodium after ligand exchange.

The original long‐chain organic ligands (oleic acid and oleylamine) passivating the surface of the quantum dots sterically stabilize the colloid. Consequently, the interaction between individual quantum dots is limited; this hinders the self‐assembly of superlattices. DFT calculations were used to explore the effect of atomic‐size ligands on the interaction between individual QDs. We first confirmed that long‐chain oleic acid molecules in the surroundings of the QD tend to bind to its surface due to a positive binding energy (Δ*E*) of 4.1 eV (**Figure** **1**a.1). Smaller inorganic ligands such as sodium ions have larger binding energies (Δ*E* = 6.4 eV), suggesting that sodium can be used to efficiently exchange the original ligand of the perovskite QDs (Figure 1b.1). We then investigated the formation energy (Δ*F*) of the superlattices by considering the interaction between individual QDs passivated with either oleic acid or sodium ions (Figure 1a.2,b.2). While the original colloid is inherently stable (Δ*F* = −7 eV), dot‐to‐dot interaction is substantially enhanced with the use of sodium ligands (Δ*F* = −16.6 eV). More details about the DFT calculations are given in the Supporting Information. A passivation strategy that results in a lower Δ*F* will slightly destabilize the colloidal solution and enable spontaneous assembly of the QDs into superlattice structures. The interparticle separation is set by the balance between ligand elastic repulsions and van der Waals attraction forces. Ligand exchange of the organic‐stabilized solution to smaller‐sized sodium ligands reduces the elastic contribution over the whole range of steric interactions, reducing the interdot distance at equilibrium.^[^ ^21^ ^]^ DFT calculations predict a threefold reduction in the interdot distance (from 10 nm with oleic acid to 3 nm with Na^+^). This is shown schematically in Figure 1a.3,b.3. In light of these findings, a ligand‐exchange approach (see the “Experimental Section”) was devised to enhance the agglomeration of QDs. The QDs form micrometer‐sized superlattice structures when precipitated from the colloidal solution (Figure 1c,d). Energy‐dispersive X‐ray spectroscopy (EDS) reveals the presence of sodium in these superlattices, confirming a successful ligand‐exchange process (Figure 1e).

### 2.2 Superlattice Microcavity Characterization

**Figure 2:**
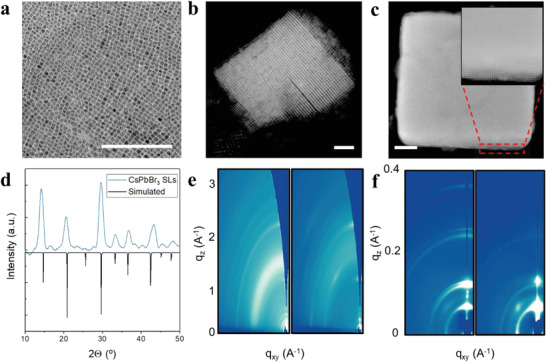
Structural characterization of SLs. TEM image of a) monodispersed QDs, b) superlattice clusters, and c) regularly shaped superlattice cube. White scale bar represents 200 nm. d) XRD pattern of SLs. The vertical lines show corresponding simulated positions. e,f) WAXS and SAXS patterns of uncoupled dots (left) and superlattices (right), respectively.

As revealed by transmission electron microscopy (TEM), the monodispersed QDs (**Figure** **2**a) self‐assemble to form cubic superlattices (Figure 2b,c). These superlattices have high packing factors as a result of the shorter distance between QDs of ≈3–4 nm (see the “Experimental Section”). This result agrees with the distance of 3 nm predicted by DFT (a 3× reduction from QDs passivated with organic ligands). The higher packing factor leads to surface‐regular superlattices in contrast to the irregular structures that form with organic‐capped dots (Figure S2, Supporting Information). This increased packing factor and the formation of atomically flat facets provide the increased optical feedback necessary for lasing.

X‐ray diffraction measurements were used to study the structural order of the superlattices. Powder X‐ray diffraction peaks (Figure 2d) correspond well with the simulated cubic structure of CsPbBr_3_. Wide‐angle X‐ray scattering (WAXS) shows localized diffraction spots arising from the short‐range spatial periodicity of the QDs in the SL structure (Figure 2e). By contrast, the non‐SL QDs show isotropic ring patterns corresponding to the absence of a favored orientation. Diffraction spots in the small‐angle X‐ray scattering (SAXS) pattern indicate that long‐range order is present throughout the structure of the superlattices, while only Debye–Scherrer rings are present for the non‐SL QD sample (Figure 2f).

### 2.3 Suppression of Auger Recombination

**Figure 3:**
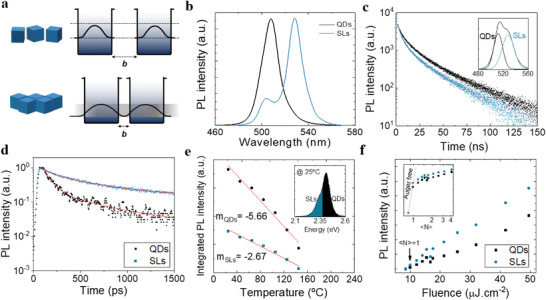
Auger recombination suppression using superlattices. a) Schematic diagram of an SL showing the delocalization of the carrier wavefunction. b) PL spectra of uncoupled QDs and SLs. c) Low‐fluence PL decay of uncoupled QDs and SLs. The inset shows the convoluted spectra formed from the emission of both coupled and uncoupled dots. d) High‐fluence PL decay of uncoupled QDs and SLs. e) Temperature‐dependent PL using a nanosecond‐pulsed laser excitation of uncoupled QDs and SLs. The inset shows the spectral range used for integration. f) Power‐dependent PL using a CW laser of uncoupled QDs and SLs. The inset shows the PL intensity as a function of the average excitonic occupancy (〈*N*〉).

We posited that, in contrast to isolated QDs, the decreased interdot distance leads to a spatial distribution of electrons and holes leading to decreased Auger recombination rates (**Figure** **3**a). The increase in carrier delocalization is shown by the PL emission of the SLs, centered around 535 nm and redshifted relative to monodispersed QDs (Figure 3b). This is achieved while retaining the identity of the individual emitters as shown by the radiative features of collective coupling (Figure 3c). At higher excitation fluences, transient PL of SLs shows a 2× increase of the radiative lifetime in comparison to uncoupled QDs (Figure 3d), with a fast lifetime component of ≈72 ps for uncoupled QDs and 135 ps for SLs (Figure S15, Supporting Information). Temperature‐dependent PL shows that the rate of decrease in PL intensity as a function of increasing temperature is greater for QDs than for SLs (Figure 3e); the SL structure better withstands the increase in temperature (lower slope in Figure 3e). Given that Auger recombination is, highly, thermally activated,^[^ ^19^ ^]^ this difference suggests that SLs suppress this nonradiative process. This analysis was performed using a nanosecond‐pulsed laser source that has a pulse duration (2 ns, 100 Hz) longer than the Auger lifetime and using a fluence of ≈30 µJ cm^−2^, which corresponds to an excitonic occupancy above 〈*N*〉 = 2.

Power‐dependent PL shows a higher rate of emission in SLs than in QDs for an excitonic occupancy above 〈*N*〉 = 1 (Figure 3f). Excitonic occupation is calculated based on the estimated absorption cross section (Figure S4, Supporting Information). The PL intensity of uncoupled QDs and SLs is normalized to the first data point, which is obtained for an average excitonic occupancy below 1 (see the “Experimental Section”). In the multibody regime (〈*N*〉 > 1), nonradiative Auger decay rates of charged excitons and biexcitons commonly outpace their radiative decay rates by orders of magnitude, resulting in extremely low emission quantum yields.^[^ ^18^ ^]^ This results in decreased photoluminescence QY of QDs with increasing excitation fluence. As shown in Figure 3f, by suppressing nonradiative Auger recombination, SLs retain a comparatively higher QY than QDs with increasing excitation fluence.

Auger recombination is associated with high exciton binding energy because of enhanced Coulombic electron–hole interaction.^[^ ^22^ ^]^ The exciton binding energy of the SLs is reduced by 30% in comparison to uncoupled QDs (Figure S11, Supporting Information), further suggesting decreased Auger recombination rate. The exciton binding energy of SLs (≈116 meV) remains higher than non‐quantum‐confined structures (Note S1, Supporting Information), therefore retaining the advantages of quantum dots in comparison to bulk perovskites (such as a higher oscillator strength).

We note that, due to focusing and signal limitations, we could not characterize Auger recombination using transient absorption. We could not probe a single superlattice directly; rather, we were limited to probing a larger area of the substrate. Thus, any signal obtained from the SLs overlapped with the overpowered signal from the surrounding uncoupled QDs.

### 2.4 Low‐Threshold Nanosecond Lasing

**Figure 4:**
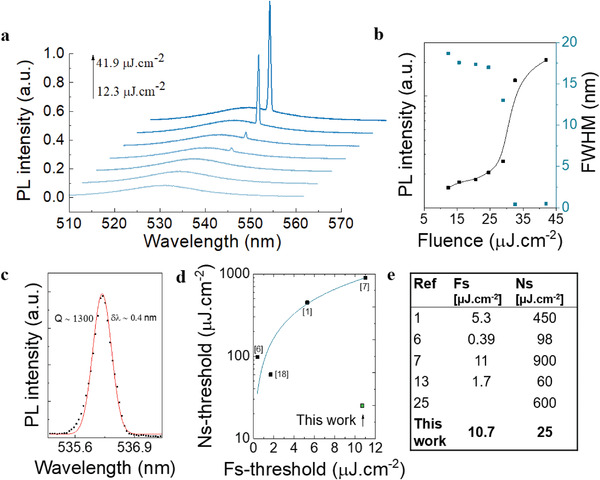
Nanosecond‐sustained lasing (1.1 ns, 15 kHz) in a CsPbBr_3_ QDs’ superlattice. a) Power‐dependent emission spectra from the SL. b) Integrated power‐dependent PL and FWHM as a function of excitation fluence, showing a lasing threshold of 25 µJ cm^−2^. c) Lasing spectra with reduced FWHM and a *Q*‐factor of 1300. d,e) Comparison of reported perovskite quantum dots’ ASE and lasing thresholds using femtosecond (fs) and nanosecond (ns) excitation.

Previously reported^[^ ^1^, ^6^, ^7^, ^13^, ^25^ ^]^ devices achieve low‐threshold lasing with femtosecond‐pulsed excitation. Likewise, low‐threshold (11 µJ cm^–2^) tunable lasing is obtained upon excitation of CsPbBr_3_ superlattices using a 400 nm femtosecond‐pulsed laser (Figure S5, Supporting Information). At longer pulse durations, above the biexciton Auger lifetime of CsPbBr_3_ QDs (of ≈25–50 ps),^[^ ^23^, ^24^ ^]^ the threshold of previously reported devices is increased by up to two orders of magnitude.^[^ ^1^, ^6^, ^7^, ^13^, ^25^ ^]^ Most works do not achieve nanosecond‐sustained lasing (Table S1, Supporting Information). Superlattices of organic‐capped dots (Figure S2, Supporting Information) do not support nanosecond‐sustained lasing and only show ASE under femtosecond‐pulsed excitation (Figure S3, Supporting Information). In contrast, the superlattices synthesized using sodium‐capped QDs—with a size of ≈2 µm (Figure S13, Supporting Information)—sustain low‐threshold lasing using nanosecond‐pulsed excitation (**Figure** **4**a).

Power‐dependent PL (Figure 4b) shows a clear threshold at ≈25 µJ cm^–2^ followed by a tenfold output‐power increase. Below threshold, there is broader PL due to spontaneous emission. By contrast, a clear full‐width at half‐maximum (FWHM) reduction (to ≈ 0.4 nm) is shown above threshold, corresponding to a resonator quality factor (*Q*‐factor) of 10^3^ (Figure 4c). The SLs sustain whispering‐gallery modes (Figure S12, Supporting Information). The best previous reports of perovskite quantum dots’ ASE and lasing with femtosecond‐pulsed and nanosecond‐pulsed excitation are combined in Figure 4d,e. In those works, Auger recombination increases the nanosecond‐sustained lasing threshold by up to two orders of magnitude from the femtosecond regime. Following the sodium ligand passivation strategy, however, nanosecond‐sustained lasing action is obtained with the minimal threshold increase, allowing us to surpass the performance of previously reported perovskite quantum dot lasers.

We report a passivation strategy that relies on sodium—an assembly director that increases the attractive forces between nearby QDs and induces the formation of long‐range ordered, regularly shaped cubic superlattices. Unlike previous superlattice works, which use organic ligands, the sodium‐rich surface of these QDs induces a process of self‐assembly to form high‐packing‐factor structures with flat facets that provide efficient optical feedback. The decreased interdot distance enables exciton delocalization that leads to decreased Auger decay. Temperature‐dependent and power‐dependent PL values confirm that nonradiative Auger recombination is better suppressed in a superlattice structure compared to uncoupled quantum dots. In contrast to previous reports, lasing action from the superlattices is relatively unchanged from the femtosecond to the nanosecond‐pulse excitation regimes, leading to low‐threshold (≈25 µJ cm^–2^) nanosecond‐sustained lasing. Ultimately, this work showcases a new strategy to suppress Auger recombination and trails a path toward the goal of continuous‐wave lasing.

## 3 Experimental Section

### Synthesis of QDs

The CsPbBr_3_ QDs were synthesized via a modified hot injection method of Dutta et al.^[^ ^26^ ^]^ Oleylammonium bromide (OLA–HBr) salt was initially prepared as follows: 10 mL of oleylamine (OAM) and 1.1 mL of hydrobromic acid (HBr) were mixed in a 50 mL three‐necked flask and heated to 40 *°*C for 30 min for degassing. Subsequently, the resulting solution was heated to 150 *°*C under the N_2_ environment and kept warm for 30 min. Simultaneously, 44.64 mg of CsCO_3_, 32.58 mg of PbO, 7 mL of octadecene (ODE), and 1 mL of oleic acid (OA) were mixed in another 50 mL three‐necked flask and degassed for 30 min at room temperature. The mixture was heated up to 230 *°*C. At this stage, 0.9 mL of OLA–HBr precursor was injected into the hot solution. The reaction was maintained for 5 min. The QDs were later precipitated in a centrifuge at 7800 rpm for 9 min. The supernatant was discarded, and the precipitated QDs were dispersed in hexane for later use.

As‐synthesized QDs (2 mL) were purified twice using 4 mL of methyl acetate (MA). The mixture was precipitated in a centrifuge at 7800 rpm for 1 min. The precipitated QDs were dispersed in 2 mL hexane.

### Ligand‐Exchange Process

For ligand exchange, 200 µL of saturated NaBr solution (2:1 toluene and dimethyl formamide (DMF)) was added to the solution of purified QDs (2 mL) and vortexed. Subsequently, 4 mL of MA was added, and the mixture was centrifuged at 7800 rpm for 1 min. The precipitated QDs were dispersed again in 2 mL of hexane. The process was repeated once more using 100 µL of the NaBr solution. Finally, the precipitated QDs were dispersed in 1 mL toluene.

### Synthesis of Superlattice Structures

After ligand exchange, the QDs were kept in a vibration‐free environment at 10 °C for 3–5 days for low‐temperature aging. From this solution, ≈500 µL of QDs was dropped on a clean MgF_2_ substrate in a saturated toluene vapor environment for 24 h with slow solvent evaporation. Superlattice structures were formed on the substrate. For superlattices with organic ligands, 500 µL of as‐synthesized CsPbBr_3_ QDs was dropped on a 1 cm × 1 cm clean MgF_2_ substrate, and then put in the same saturated toluene vapor environment for 24 h.

### Structural Characterization

X‐ray diffractograms were recorded using a Rigaku MiniFlex 600 powder X‐ray diffractometer equipped with a NaI scintillation counter and using monochromatized Cu K*α* radiation (*l* = 1.5406 Å °). WAXS and SAXS measurements were conducted at the Canadian Light Source (CLS). A lead beamstop was used to block the direct beam. Images were processed via the Nika^[^ ^27^ ^]^ software package and the GIXSGUI^[^ ^28^ ^]^ MATLAB plugin.

TEM analysis was carried out on a Hitachi HF 3300 operating at a beam energy of 300 keV. ImageJ^[^ ^29^ ^]^ was employed to determine the size distribution of the QDs. EDS analysis was performed using a field‐emission scanning electron microscope (Auriga S40, Zeiss, Oberkochen, Germany). The black spots in the EDS mapping (Figure 1e) were attributed to charging, which was resulted from 1) the substrate of the sample in glass and 2) the left‐over of organic residues that remained on the substrate from the sample preparation process. By exposing the sample to high voltages during long periods of time, the volatilization of the sample may occur leading to black spots on the EDS pattern. The interdot distance was estimated based on the fast Fourier transformation (FFT) of the TEM images. The superlattice periodicity (corresponding to the interdot distance) results in points in the frequency domain, which were used to estimate the average distance between QDs.

### Optical Characterization

PL and single‐mode lasing spectra measurements in Figure S5 (Supporting Information) were performed on a 400 nm femtosecond‐pulsed laser (Libra, Coherent, 40 fs, 10 kHz) with a confocal micro‐photoluminescence system (LabRAM HR Evolution). Time‐resolved PL measurements were recorded with a Horiba Fluorolog correlated single‐photon counting detector using a 374 nm nanosecond‐pulsed laser. Temperature‐dependent analysis was performed using a variable temperature pourfill (VPF) liquid nitrogen cooled cryostat (Janis Research). Samples were optically excited using a 355 nm frequency‐tripled Nd:YAG laser with a pulse width of 2 ns and a repetition rate of 100 Hz.The spectral data were collected using a UV–vis USB 2000+ spectrometer (Ocean Optics). The data given in Figure 3f were obtained using the 355 nm frequency‐tripled Nd:YAG laser with a pulse width of 2 ns and a repetition rate of 100 Hz. It was difficult to measure directly PLQY due to the small size of the SLs, as such; instead, the relative change in PL intensity was compared with increasing fluence. Power‐dependent measurements were performed on uncoupled QD and SL samples using an excitation fluence range that leads to an average excitonic occupancy, 〈*N*〉, below and above 1. Then the results were normalized using the PL intensity value obtained for the first data point (〈*N*〉 below 1) for both uncoupled QDs and SLs. This way the relative change in PL intensity was analyzed with increasing fluence. The data given in Figure 3d were obtained by using a streak camera (Hamamatsu C10910) with 400 nm excitation (80 fs, 1 kHz repetition rate). Nanosecond excitation used for the measurements shown in Figure 4 was performed with a Nd:YAG laser with a pulse width of 1.1 ns and a repetition rate of 15 kHz (2Q‐STA‐IL, Germany).

### Density Functional Theory

In the computational method, electronic structure calculations were performed in the framework of DFT. Detailed information is given in the Supporting Information.

### Statistical Analysis

The data were used without any transformation with the exception of the following; in Figures 3b,d and 4a the spectra were normalized; in Figure 3f, the integrated PL was normalized as described in this section. Statistical analysis was performed, as shown in Figures S4 and S13 (Supporting Information), to determine the size of QDs and SLs. The mean value, standard deviation, and sample size were provided in the corresponding caption.

## Conflict of Interest

The authors declare no conflict of interest.

## Supporting information

Supporting Information

## Data Availability

The data that support the findings of this study are available from the corresponding author upon reasonable request.
