# Supplementary material for: Quantum Dot Self‐Assembly Enables Low‐Threshold Lasing
Source: Adv Sci (Weinh). 2021 Aug 27;8(20):2101125. doi: 10.1002/advs.202101125 (PMC8529423; doi:10.1002/advs.202101125)
Supplement: Supplementary file 1 — Supporting Information [file ADVS-8-2101125-s001.pdf]

© 2021 Wiley-VCH GmbH

ADVANCED SCIENCE
Open Access

Supporting Information

for *Adv. Sci.,* DOI: 10.1002/advs202101125

Quantum dot self-assembly enables low-threshold lasing

Chun Zhou, Joao M. Pina, Tong Zhu, Darshan H. Parmar, Hao Chang, Jie Yu, Fanglong Yuan, Golam Bappi, Yi Hou, Xiaopeng Zheng, Jehad Abed, Hao Chen, Jian Zhang, Yuan Gao, Bin Chen, Ya-Kun Wang, Haijie Chen, Tianju Zhang, Sjoerd Hoogland, Makhsud I. Saidaminov, Liaoxin Sun, Osman M. Bakr, Hongxing Dong,* Long Zhang* and Edward H. Sargent*

Supporting Information

Quantum dot self-assembly enables low-threshold lasing

Chun Zhou[1,2,4+], *Joao M. Pina*[2+], *Tong Zhu*[2+], *Darshan H. Parmar*[2], *Hao Chang*[1,4], *Jie Yu*[1,4], *Fanglong Yuan*[2], *Golam Bappi*[2], *Yi Hou*[2], *Xiaopeng Zheng*[3], *Jehad Abed*[2], *Hao Chen*[2], *Jian Zhang*[5], *Yuan Gao*[2], *Bin Chen*[2], *Ya-Kun Wang*[2], *Haijie Chen*[2], *Tianju Zhang*[1], *Sjoerd Hoogland*[2], *Makhsud I. Saidaminov*[2,6], *Liaoxin Sun*[5], *Osman M. Bakr*[3], *Hongxing Dong*[1*], *Long Zhang*[1*] and *Edward H. Sargent*[2*]

Supporting information

Figure S1 – PL spectra of isolated dots and SLs

Figure S2 – SEM images of superlattices with organic ligands

Figure S3 – ASE in superlattices with organic ligands

Figure S4 – Estimation of excitonic occupation

Figure S5 – Femtosecond lasing of SLs

Figure S6 – DFT calculation of the crystal structure of atomic ligand (Na^+) treated $CsPbBr_3$ surface

Figure S7 – DFT calculation of the crystal structure of organic ligand (oleic-acid) treated $CsPbBr_3$ surface

Figure S8 – DFT calculation of the interactions between the atomic ligand (Na) treated $CsPbBr_3$ surface and a nearby $CsPbBr_3$ surface

Figure S9 – DFT calculation of the interaction between the clean $CsPbBr_3$ surface and a nearby $CsPbBr_3$ surface

Figure S10 – SL cavity size and single-mode lasing

Figure S11 – Decreased binding energy

Figure S12 – Whispering-gallery mode lasing

Figure S13 – SL size distribution

Figure S14 – Lasing spectra before and after lasing threshold

Table S1 – ASE and lasing characteristics of perovskite quantum dots

Note S1 – Balance between quantum confinement and Auger recombination

Note S2 – Carrier transport with inorganic ligands

Note S3 – Carrier dynamics under femtosecond and nanosecond optical excitation

Figure S1

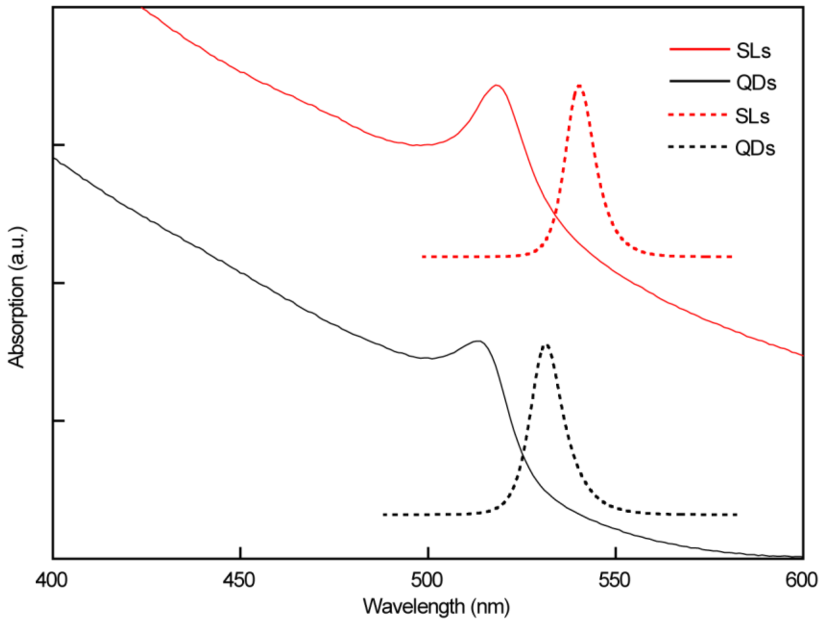

Fig S1: Absorption and PL spectra of QDs and SLs.

Figure S2

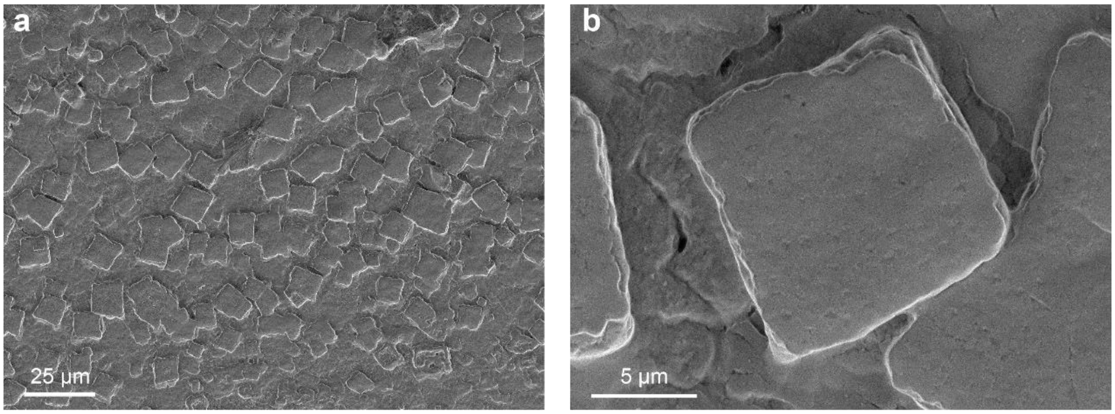

Fig S2: SEM images of superlattices with organic ligands. a-b QDs with organic ligands self-assembled to cubic superlattices with rough edges and surfaces.

Under femtosecond-pulsed excitation, superlattices with inorganic ligands and superlattices with organic ligands show traces of optical gain (ASE and lasing). The threshold for optical gain is higher in SLs (lasing at P_{th}=11 μJ.cm^{-2}, Figure S5b) in comparison to QDs with organic ligands (ASE at P_{th}=4.1 μJ.cm^{-2}, Figure S3) presumably because of factors such as the lower oscillator strength (Note S3).

However, once using nanosecond-pulsed excitation, SLs formed with inorganic ligands sustain lasing at low thresholds. On the other hand, SLs formed with organic ligands do not show any traces of optical gain. This suggests that non-radiative processes that are not triggered in the femtosecond time-scale but are triggered in the nanosecond regime, such as Auger recombination, quench lasing/ASE in SLs with organic ligands but not in SLs with inorganic ligands.

Figure S3

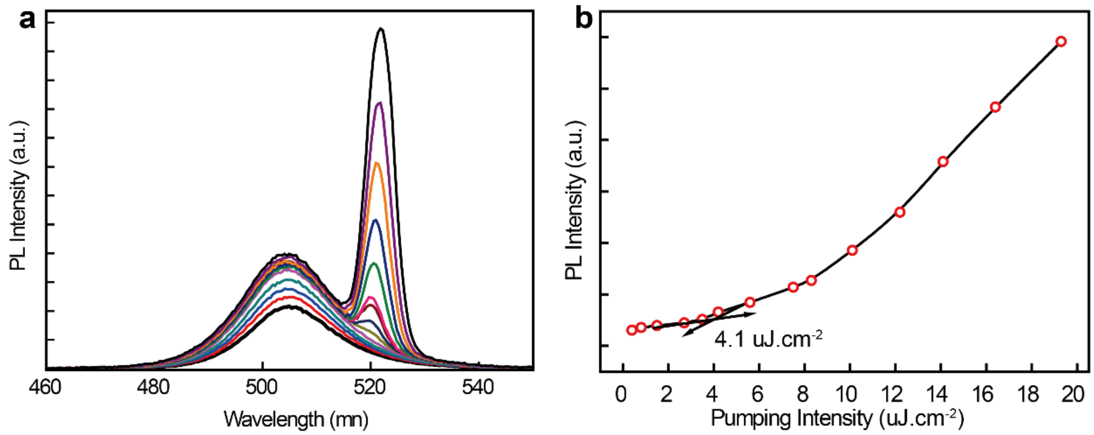

Fig S3: ASE in superlattices with organic ligands. a Pump power-dependent ASE from a single $CsPbBr_3$ superlattice with organic ligands using a 350 nm fs-laser (5000 Hz). **b** Integrated PL as a function of pump density showing ASE threshold at 4.1 μJ.cm^{-2}. The SLs do not show ASE/lasing when increasing the pulse duration to the nanosecond scale.

Figure S4

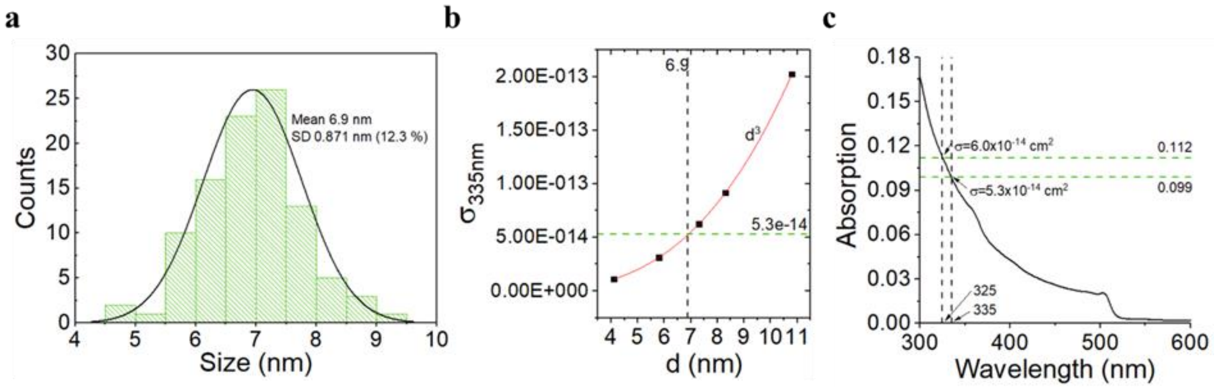

Fig S4: Auxiliary information used to determine excitonic occupancy as a function of fluence. a Histogram of QD size based on the TEM of Fig. 1b obtained for a population of 100 QDs. **b** Cross-section (σ_{335nm}) estimation at 335 nm based on previously reported[1] values. **c** Absorption spectrum of $CsPbBr_3$ QDs.

Excitonic occupation (Fig. 3e) was determined based on the estimated absorption cross-section. The average QD size is determined to be of 6.9 nm (Fig. S4a), consistent with the synthesis conditions used (see Methods). Based on previously reported cross-section values of $CsPbBr_3$ QDs at 335 nm[1] and the average diameter of 6.9 nm, the absorption cross-section is estimated to be around 5.3×10^{-14} cm^2 (Fig. S4b). The absorption spectrum of $CsPbBr_3$ is used to scale the estimated absorption cross-section from 335 nm to 325 nm (Fig S4c), the excitation wavelength used during the power-dependence analysis shown in Fig. 3e. The average excitonic occupancy is determined based on the relation shown below.

$$< N > = \frac{Fluence \times Cross\ section}{Photon\ energy}$$

Figure S5

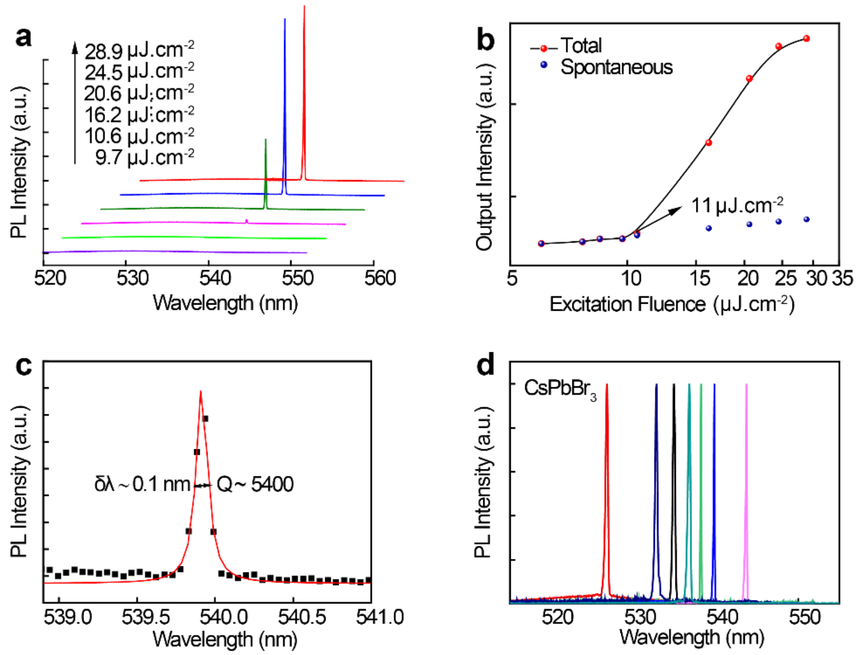

Fig. S5: Tunable femtosecond single-mode lasing in $CsPbBr_3$ QDs superlattice microcavities. a Pump power-dependent single-mode lasing from a single $CsPbBr_3$ superlattice using a 40 fs-pulsed excitation. **b** Integrated PL as a function of pump density showing lasing threshold at 11 μJ.cm^{-2}. **c** Lorentz fitting of a lasing mode. The corresponding FWHM is ~0.1 nm corresponds to a quality mode factor of ~5400. **d** Single-mode lasing spectra of seven $CsPbBr_3$ superlattices with different edge-length.

DFT calculations methodology

All the DFT calculations were performed using the FHI-aims (1,2,3) all-electron code. The default numerical settings, referred to as "intermediate" in FHI-aims, were used. Local minimum-energy geometries of the Born-Oppenheimer surface were obtained with residual total energy gradients below $1x10^{-2}$ eV/ Å for atomic positions by PBE-GGA functional within the vdW correction following the TS approach (PBE+TS). We begin from the geometry relaxation of the cubic unit cell for $CsPbBr_3$ (*Pm-3m* space group) by PBE+TS with k-grid 6x6x6. Then, this PBE+TS relaxed unit-cell is used to build a slab with 3x3x4 supercells. The total length at the c-direction (including the vacuum area) is 200 Å. The bottom layer of the slab is passivated by hydrogen atoms in order to replicate the chemical environments of the bottom bulk. The bond length of the hydrogen bonding is obtained by PBE+TS relaxation of the whole slab structures (k-grid is set to 2x2x1) with fixed positions for all the Cs, Pb or Br atoms.

1. In order to obtain the binding energies ($E_{binding}$[ligands] = E_{tot}[$CsPbBr_3$] + E_{tot}[ligands] - E_{tot}[$CsPbBr_3$ with ligands]) of different ligands (atomic ligands, Na, and organic ligands, oleic-acid), three different initial configurations are considered in which those ligands are attached on top of the Cs, Pb or Br atoms (as shown in Fig. S6/S7 for Na/Oleic-acid ligands). Then, those initial structures are also relaxed by PBE+TS within k-grid 2x2x1 (the bottom two layers are connected to hydrogen atoms which are fixed in order to replicate the effects from the bottom bulk). For atomic ligand Na (Fig. S6), we find that the Na atom prefers to settle in the outmost exposure surface formed by Pb and Br atoms (within almost the same c-direction values), and on top of the below Cs atoms after the relaxations. The binding energies calculated from these three different initial configurations are similar (around 6.4 eV). For organic ligand oleic-acid (Fig.

S7), we find that the oleic-acid prefers to attach with the Pb atoms after the relaxations. The associate binding energies calculated from these three different initial configurations are also similar, of around 4.0 eV.

2.To compare the effects of the different ligands on the self-assembly of $CsPbBr_3$ quantum dots, we calculate the formation energies (ΔF) defined as follows:

$$\Delta F = E_{tot}(all) - E_{tot}(bottom) - E_{tot}(top)$$

Where E_{tot}(all) represents the total energy of the whole structure (Fig. S8), the E_{tot}(bottom) represents the total energy of the ligand-treated surface in the bottom part, and the E_{tot}(top) represents the total energy of the clean surface in the top part. For the ligand-treated surface (bottom structures), as their binding energies are almost the same, structures that relaxed from Na/Oleic-acid on top of Br/Pb atom are used for Na/Oleic-acid ligand. For the clean surfaces (top structures), two different surfaces are considered – the surface formed by Pb, Br atoms, and Cs, Br atoms (Fig. S8 and S9). The exposed surfaces formed by Cs, Br atoms have the lowest formation energies. The formation energy, -10.10/-16.57 eV, of atomic ligand (Na) treated-surface for the exposed-surface formed by (Pb, Br)/(Cs, Br) is significantly larger than that of organic ligand tread-surface (-2.54/-6.99 eV).

Figure S6

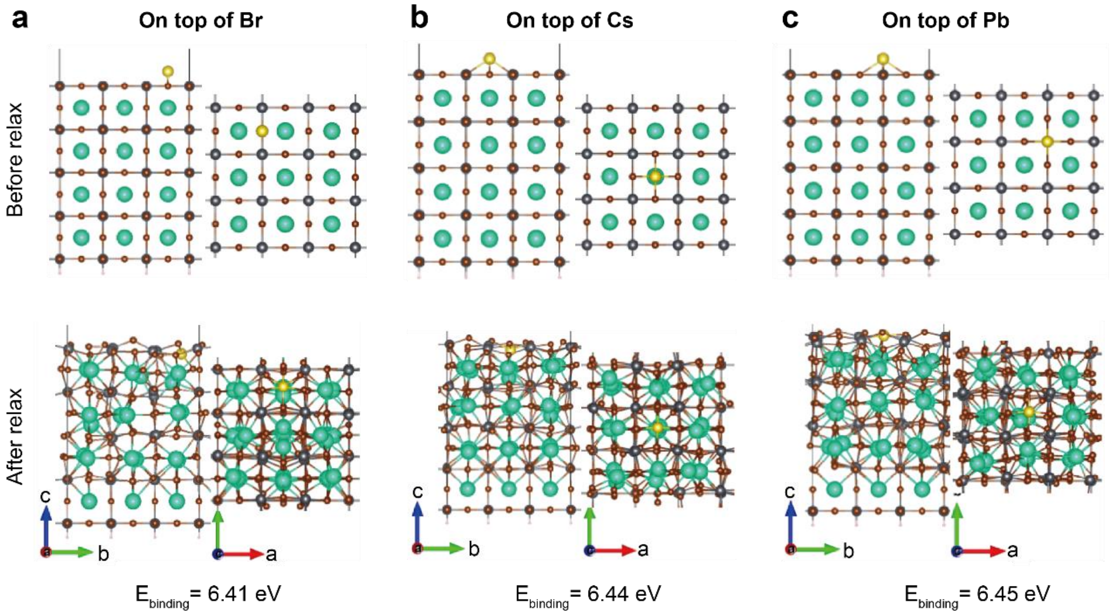

Figure S6. Crystal structure of atomic ligand (Na) treated $CsPbBr_3$ surface. The sodium atom is set above different surface regions (on top of Br, Cs and Pb) before relaxation. The structure is then relaxed to obtain the associated binding energy of a sodium atom to the surface of $CsPbBr_3$. The Cs, Pb, Br, Na, and H are shown in cyan, grey, brown, yellow, and pearl color, respectively.

Figure S7

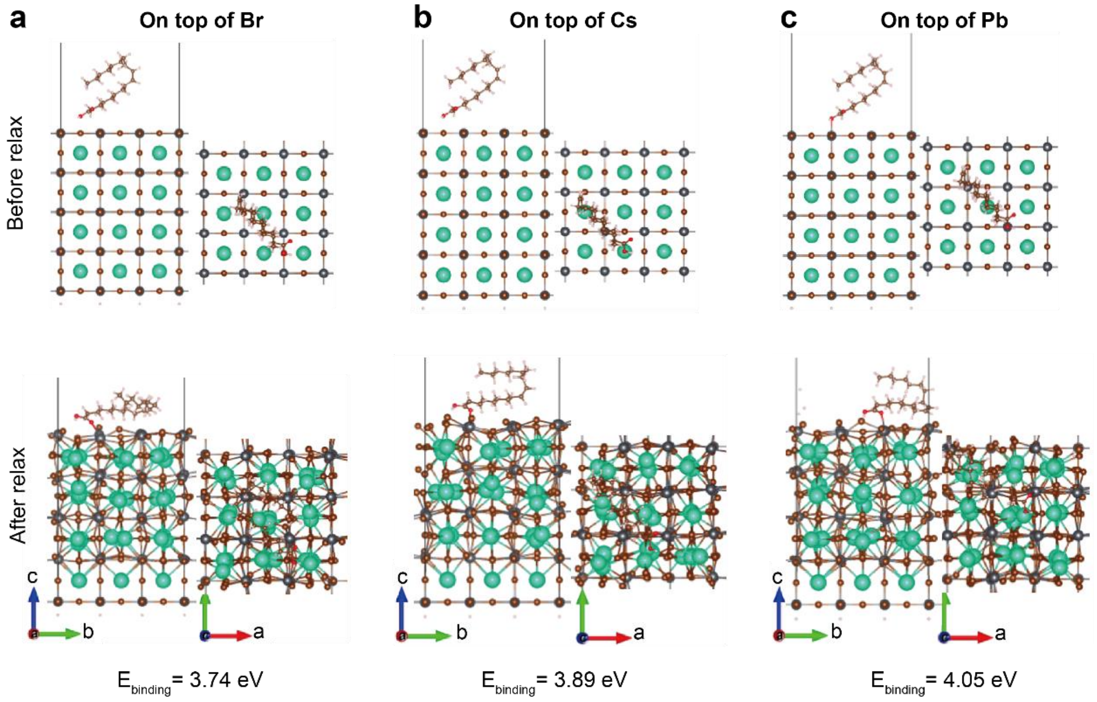

Figure S7. Crystal structure of organic ligand (oleic-acid) treated $CsPbBr_3$ surface. The oleic-acid is set above different surface regions (on top of Br, Cs and Pb) before relaxation. The structure is then relaxed to obtain the associated binding energy of an oleic-acid molecule to the surface of $CsPbBr_3$. The Cs, Pb, Br, C, O, H are shown in cyan, grey, brown, light brown, red, and pearl color, respectively.

Figure S8

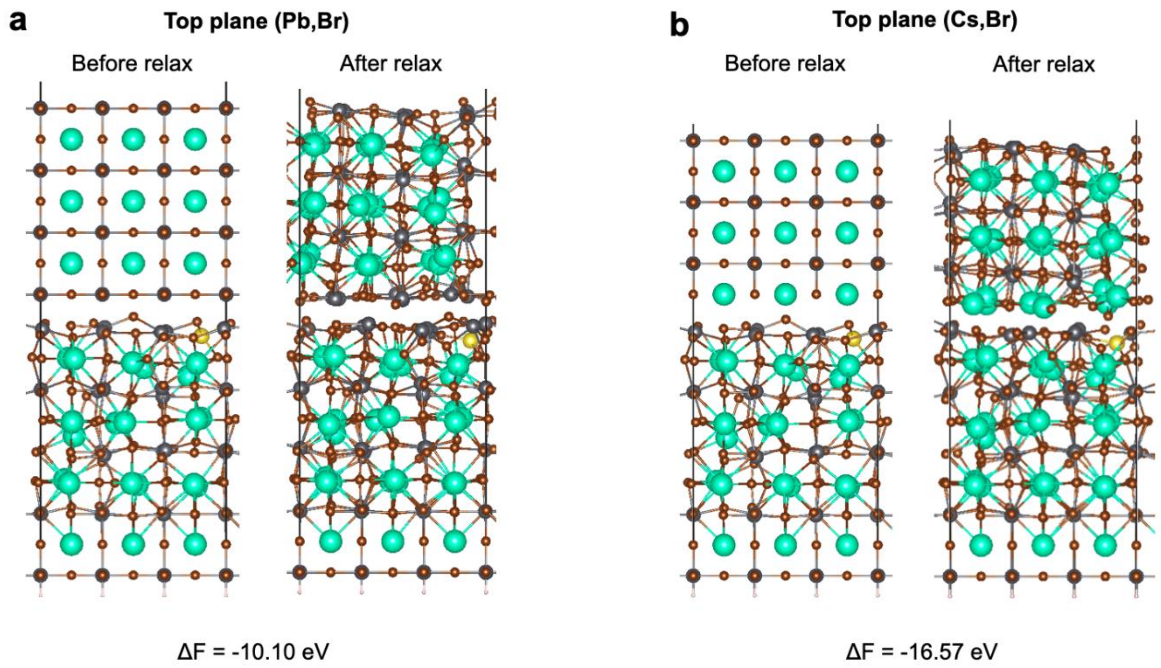

Figure S8. Interaction between the atomic ligand (Na) treated $CsPbBr_3$ surface and a nearby $CsPbBr_3$ surface (the exposure plane is formed by (Pb,Br)/(Cs,Br) atoms). The Cs, Pb, Br, Na, H are shown in cyan, grey, brown, yellow, and pearl color, respectively.

Figure S9

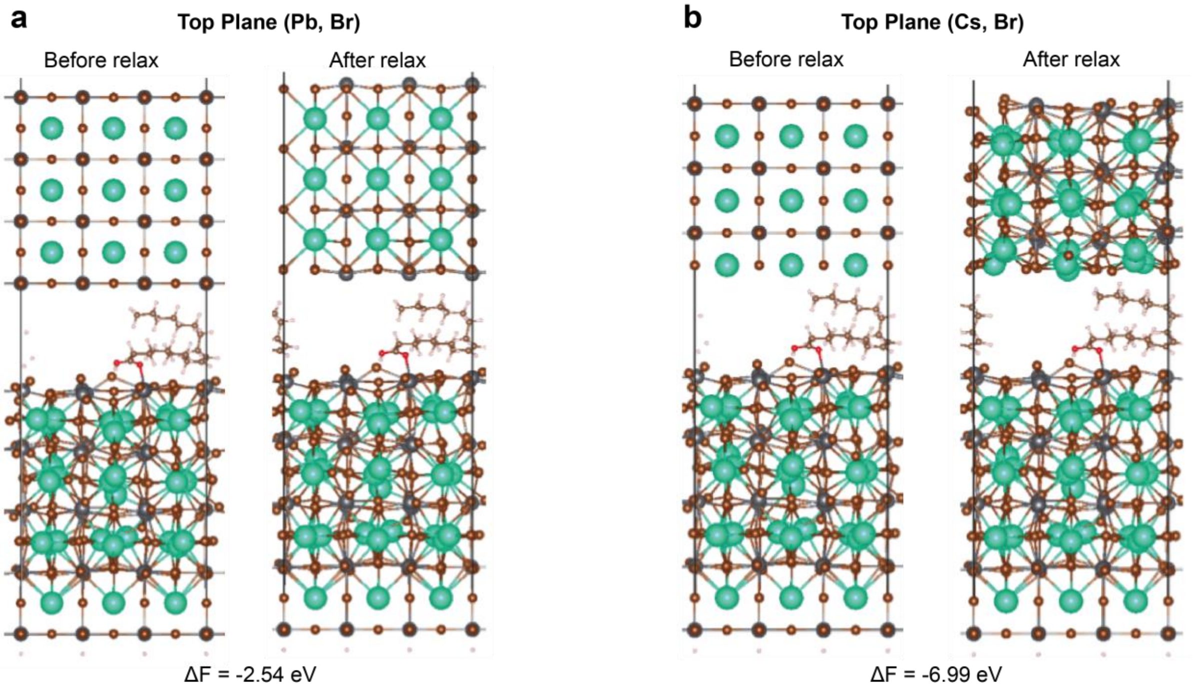

Figure S9. Interaction between the clean $CsPbBr_3$ surface and a nearby $CsPbBr_3$ surface passivated with organic ligands (the exposure plane is formed by (Pb,Br)/(Cs,Br) atoms). The Cs, Pb, Br, H are shown in cyan, grey, brown, and pearl color, respectively.

Figure S10

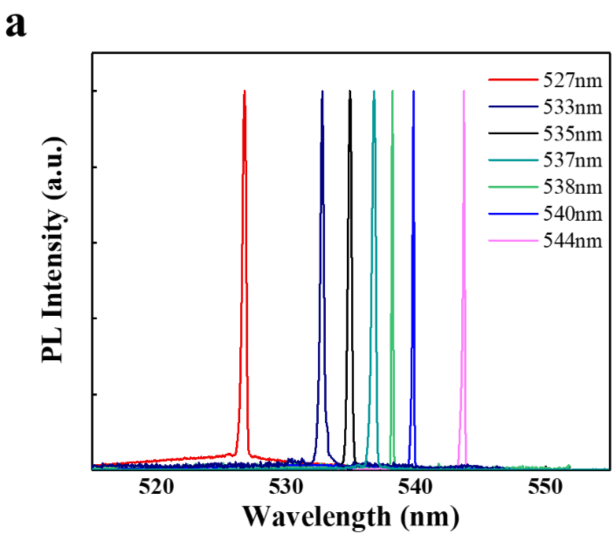

Figure S10. Impact of cavity-size on SL lasing. **a** Single-mode lasing spectra of seven $CsPbBr_3$ superlattices with different edge-length.

Assuming an optical resonator consisting of two sets of mirrors where light is reflected with normal incidence on both mirrors, we have the following resonance relation:

$$l \times \left(\frac{\lambda}{n}\right) = 2 \times L$$

Where λ is the wavelength of light, *n* is the refractive index of the medium and *l* is an integer.

The refractive index of the SLs should be of around 1.9-2. The cavity length of SLs is of around 2 (size distribution provided in Figure S13). The lasing wavelength we have obtained in Figure 4 is around 536 nm. This leads us to the cavity mode of *l* ~ 15. For cavity mode *l* = 14 and *l* = 16 (the modes that resonate at the closest wavelengths to 536 nm), we obtain $\lambda_{l=14}$=571 nm and $\lambda_{l=16}$=500 nm. Both modes are outside of the gain bandwidth of the laser, which is limited by the gain medium and it is of 7-15 nm judging by the ASE width (Fig. S3). The single lasing peak is tunable from around 527 nm to 544 nm (within the gain bandwidth) by slightly varying the cavity length. It is important to note that we do not have such precise synthetic control – the synthesis leads to SLs with a range of sizes.

Figure S11

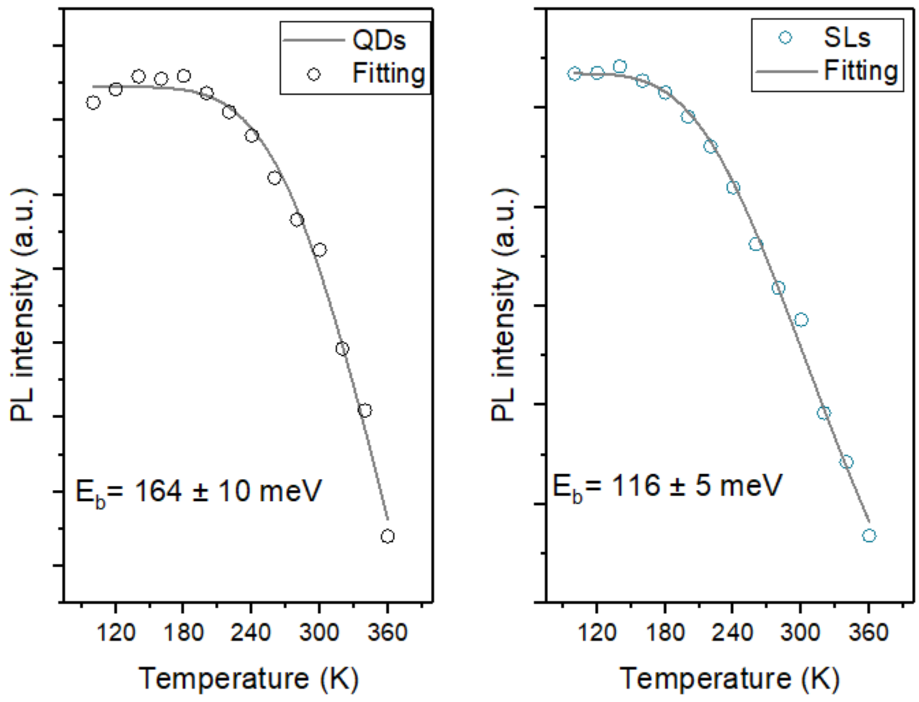

Figure S11. Temperature-dependent integrated photoluminescence (PL) intensity at different temperatures for QDs and SLs.

The exciton binding energy was evaluated by the temperature dependence of the integrated PL signal and fitted by the following Arrhenius equation[5,6]:

$$I(T) = \frac{I_0}{1 + A \times e^{(\frac{-E_b}{k_B \times T})}}$$

where I_0 is the integrated PL intensity at 0 K, A is a constant, E_b the binding energy and kb the Boltzmann constant. Since the PL intensity flats out for lower temperatures, I_0 at 0 K is extrapolated from the 120K-180K where intensity remains constant.

This method can be used when assuming that depopulation processes are dominated by thermal dissociation and radiative spontaneous emission processes[5]. The decrease in PL with increasing temperature is due to the increase in thermal dissociation rate of excitons at high temperatures[5]. This is a reasonable assumption considering the high PLQY (> 80%) of the material system at the low fluence (<N> < 1) used in this analysis.

Auger recombination is associated with high exciton binding energy because of enhanced Coulomb electron-hole interaction. A higher binding energy leads to less uniformly distributed carriers in space, therefore increasing the multi-body interaction probability (two electrons and

one hole at the same position) and accelerating Auger recombination[6,7]. In one-dimensionally confined materials, the Auger recombination rate is proportional to the third power of binding energy[6,8]. Therefore, reducing the binding energy leads to a decrease in Auger recombination in quantum-confined systems[6].

Figure S11 shows that the binding energy is decreased by 30 % from the uncoupled QDs to SLs. This is due to the decreased inter-dot distance (as shown in Fig. 1 and Fig. 2, inter-dot distance is reduced from 10 nm to 3 nm in a SL), which enables exciton delocalization and leads to decreased Auger recombination rates.

It is important to note that decreasing E_b also decreases first-order exciton recombination due to trap-assisted nonradiative recombination[6]. However, lasing at the nanosecond regime is not limited by trap-assisted recombination but by Auger recombination; therefore, decreasing Auger recombination will improve lasing operation.

Figure S12

a) b)

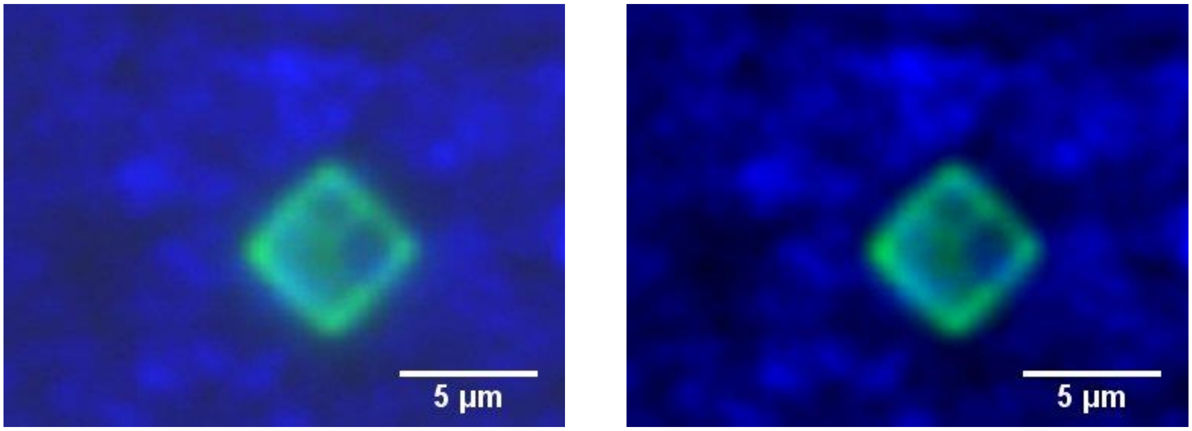

Figure S12. Emission at the four corners of the microcavity suggests the occurrence of in-plane WGM lasing.

Attenuated microscope image of SL lasing without a) and with b) increased contrast.

The work by Li, Qi et al.[9] suggests that Fabry-Perot (FP) modes are only present for large edge length microplatelets (above 13 µm). Our superlattices have edge length of around 2-6 µm suggesting whispering-galley-modes (WGM). As in the work of Li et al., the occurrence of stronger emission at the four corners of the microcavity suggests the occurrence of in-plane WGM cavity lasing.

Figure S13

a)

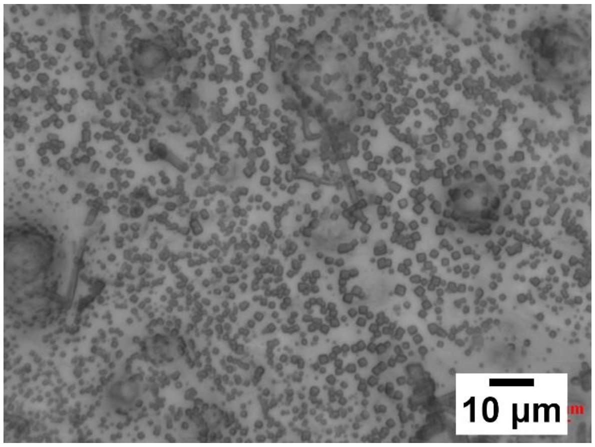

b)

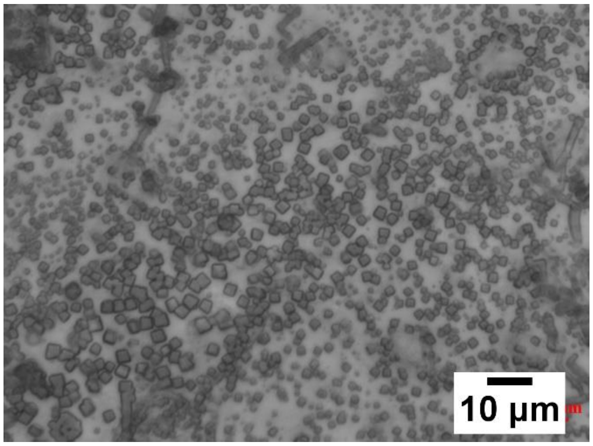

c)

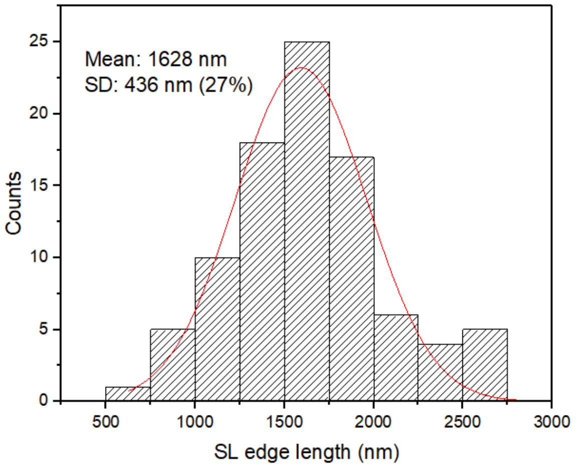

Figure S13. Size distribution of SLs. **a-b** Microscope images of SLs and **c** size distribution obtained for a population of 91 SLs (taken from Figure S13a and S13b, approximately 50-50%).

Figure S14

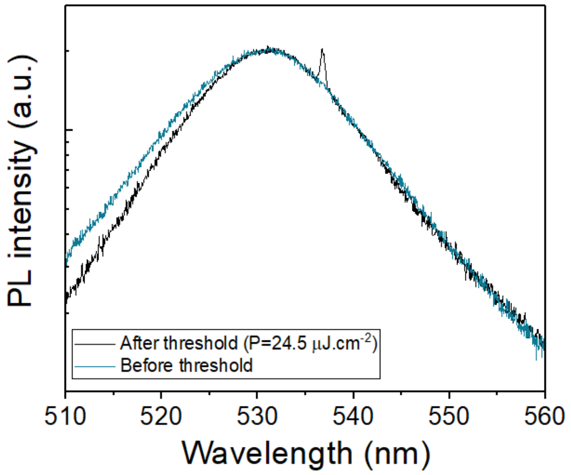

Figure S14. PL spectra before and after threshold, showing the appearance of a lasing peak at a fluence of 24.5 μJ.cm^{-2}.

Figure S15

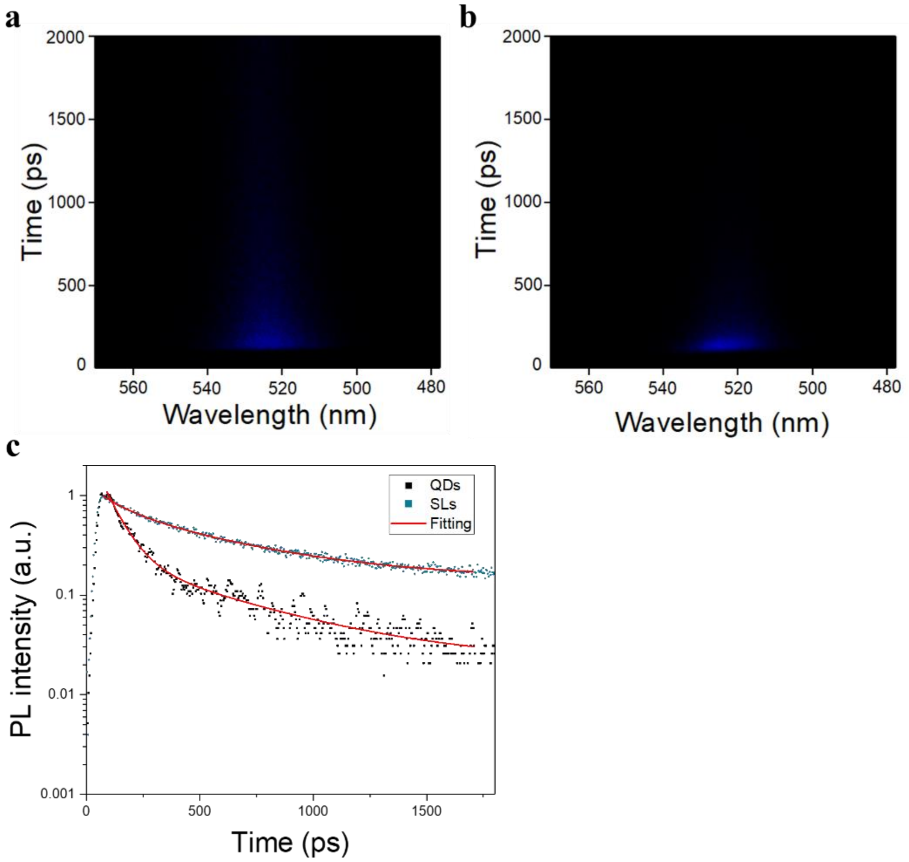

Figure S15. Time-resolved PL of **a** SLs and **b** QDs at a fluence of 0.9 μJ.cm^{-2} and 1.2 μJ.cm^{-2}, respectively. The time-trace is fitted in **c**.

To assess the properties of multiexciton states, we excite the uncoupled QDs and SLs with 80 fs pulses (1 kHz repetition rate) using a wavelength of 400 nm. Figure S15 shows an example of time-resolved PL obtained using a streak camera (Hamamatsu C10910). At low excitation fluence, time-resolved PL shows the typical single-exciton recombination lifetime of tens-of-ns (as shown in Figure 3c). When increasing the excitation fluence, a much faster time component (tens-of-ps) emerges. This is a signature of the generation of multiple excitons which decay via Auger recombination [10].

Uncoupled QDs show a much stronger Auger recombination rate in comparison to SLs (Figure S15). Fitting yields a fast lifetime component of approximately 72 ps for uncoupled QDs and 135 ps for SLs. We attribute the improvement to the delocalization of charge carriers in the SL structure.

We note that both uncoupled QDs and SLs show traces of optical gain (ASE in uncoupled QDs [Figure S3] and lasing in SLs [Figure S5]) under femtosecond excitation. The fluence used for this analysis was chosen by increasing the excitation power until traces of a faster time component emerged. This was sufficient for direct comparison. Using higher fluences –

approaching that of optical gain – may reduce lifetime due to accelerated radiative emission due to stimulated emission. We note that although the analysis was conducted with the same fluence for SLs and uncoupled QDs (and therefore, similar <N>), QDs are closer to ASE threshold ($0.3P_{th}$) than SLs ($0.1P_{th}$). The small differences between the fluence used for QDs and SLs arise from limitations controlling the laser power.

Table S1

Table S1: ASE and lasing characteristics of representative quantum-confined perovskite structures. As shown, reports of nanosecond-sustained stimulated emission remain few. NWs stands for nanowires.

Material	Femtosecond-threshold (μJ/cm^2)	Femtosecond pulse duration	Nanosecond-threshold (μJ/cm^2)	Nanosecond pulse duration	Reference
**$CsPbBr_3$ QDs**	**11**	**40 fs**	**25**	**1.1 ns**	**This work**
$CsPbBr_3$ QDs	5.3	100 fs	450	10 ns	Nat. Commun. 2015, 6, 8056
$CsPbBr_3$ QDs	0.39	50 fs	98	5 ns	ACS Photonics 2017, 4, 9, 2281-2289
$CsPbBr_3$ QDs	11	100 fs	900	5 ns	Adv. Funct. Mater.2017, 1605088
$CsPbBr_3$ QDs	1.7	150 fs	60	5.5 ns	ACS Appl. Mater. Interfaces 2018, 10, 47, 40661-40671
$CsPbBr_3$ QDs	192	35 fs			J. Phys. Chem. Lett. 2015, 6, 24, 5027-5033
$CsPbBr_3$ QDs	22	100 fs			Adv. Mater. 2015, 27, 7101–7108
$CsPbBr_3$ NWs	6.2	150 fs			ACS Nano 2016, 10, 8, 7963-7972
$CsPbI_3$ QDs	50	100 fs			Adv. Mater. 2015, 27, 7101–7108
$CsPbI_3$ QDs	17	100 fs			Nat. Commun. 2015, 6, 8056
$CsPbI_3$ NWs	6	150 fs			ACS Nano 2016, 10, 8, 7963-7972
$CsPbCl_3$ NWs	6	150 fs			ACS Nano 2016, 10, 8, 7963-7972
$MAPbBr_3$ QDs	13	150 fs			Nano Lett. 2017, 17, 12, 7424-7432
$MAPbI_3$ NWs	0.22	150 fs			Nat. Materials 2015, 14, 636-542
$MAPbI_3$ platelets	40	50 fs			Nano Lett. 2014, 14, 5995-

					6001
$FAPbBr_3$ NWs	7	100 fs			Nano Lett. 2016, 16, 2, 1000-1008
$(OA)_2(MA)_{n-1}Pb_nBr_{3n+1}$ platelets	8	150 fs			Adv. Mater. 2018, 30, 1707235
MAPbX3 QDs	This work reports CW lasing with a threshold of 15 W/cm^2.				J. Phys. Chem. Lett. 2019, 10, 12, 3248–3253

Note S1

In this note, we discuss the balance achieved between quantum confinement and reduced Auger recombination. Data suggests that the constituents of the laser are still quantum dots, and therefore, the laser still benefits from the advantages of using quantum dots such as a higher oscillator strength that arises from quantum confinement.

As shown in Figure S11, the exciton binding energy of the superlattices (116 meV) is smaller than that of strongly quantum-confined perovskites quantum-dots and nanoplatelets such as:

162–272 meV for QDs and 32 meV bulk film (Journal of colloid and interface science 539 (2019): 619-633.)

120 meV (The journal of physical chemistry letters 8.6 (2017): 1161-1168)

320 meV for QDs (The journal of physical chemistry letters 6.15 (2015): 2969-2975.)

But it is significantly higher than that of perovskite single and polycrystalline samples:

37 meV (Journal of Luminescence 226 (2020): 117471)

16 meV (The journal of physical chemistry letters 8.8 (2017): 1851-1855)

27.3 meV (Optics express 27.20 (2019): 29124-29132)

Reports of the exciton binding energy for quantum confined perovskites are usually within a range of 100-350 meV, and for bulk perovskites within 10-40 meV.

In contrast to bulk perovskites, where the low PLQY is often attributed to the low exciton binding energy (10-40 meV) at room temperature (kT~26 meV), our superlattice material retains a much higher binding energy (116 meV). This higher binding energy shows that in contrast to structures without quantum confinement, the SL material still retains the advantages of quantum dots (such as the high exciton binding energy and higher oscillator strength).

Nevertheless, the material has a lower excitonic binding energy than the original quantum-dots (Figure S11). This suggests that the material indeed has reduced Auger recombination, therefore striking a balance between the advantages of quantum-confinement and the disadvantages of Auger recombination.

Note S2

This work showcases a new strategy to suppress Auger recombination and trails a path forward towards the ultimate goal of lasing with direct-current electrical pumping. Under femtosecond pulsed excitation, Auger recombination plays a minimal role since the Auger process is significantly slower (in the order of 50 ps) than the formation of optical gain. Extending the duration of lasing in perovskite QDs to the nanosecond and further on to the microsecond time range, which is significantly superior to that of biexciton Auger lifetime, is, therefore, an important step towards direct-current electrical pumping. The use of superlattices offers three potential benefits (self-assembly of a lasing cavity, decreased non-radiative recombination processes, and improved carrier transport) towards this goal which can only be achieved with optimization in terms of electrical and optical properties.

This work reports the use of short-chain ligands (alkali-metals) to substitute the long-chain organic ligands that traditionally passivate the surface of perovskite QDs. Large advances in QD optoelectronics have required the substitution of the long organic ligands with shorter ligands, providing increased QD coupling and improved charge transport[11]. The ligand-exchange reported in this work is likely to improve charge transport in the material system – as reported for the case of LEDs – although the advantage will only be applicable with advances in synthetic control that lead to the formation of SL films.

Ligand-exchange of perovskite quantum-dots has led to record high quantum-efficiency LEDs. Wu, T. et al.[12] use a lithium halide passivation on perovskite QDs to achieve a high brightness of 50 270 cd.m^{-2}. Dong, Y. et al.[13] later reported the use of sodium bromide and $C_3H_{10}BrN$, showcasing an 100x improvement in carrier mobility in comparison to reports of QDs with organic ligands. Wang, Y. et al.[14] used potassium halides achieving high-performance (23 % EQE) and stability (T50 of 10h at 200 cd.m^{-2}).

Note S3

We compare the carrier dynamics of the SLs under excitation with femtosecond (40 fs, 10 kHz) and nanosecond (1.1 ns, 15 kHz) optical excitation. The analysis here provided follows Qin, J. et al.[15].

Under femtosecond-pulsed excitation, the pulse time (τ_{pulse}) is much shorter than the carrier lifetime ($\tau_{pulse} << \tau_{carrier}$). As such, the carrier recombination rate is negligible compared with the carrier generation rate (G) and the carrier density is attributed only to the carrier generation.

The carrier density (n) is therefore calculated based on Equation 1:

$$n = (1 - R)(1 - e^{-\alpha d}) \frac{I_{fs}}{d \frac{hc}{\lambda}}$$

Where R is the reflectance (R~10%), α is the absorption coefficient (5x10^{4} cm^{-1}), d is the thickness of the SLs (d~2 µm; due to the large size of the SLs, we use the penetration depth, L = 1/ α), I_{fs} is the laser fluence (J.cm^{-2}) of the excitation at wavelength λ (400 nm), h is Plank's constant, and c is the speed of light. This relation is applicable for femtosecond and picosecond laser excitation.

In Figure S15a, we calculated the carrier density under femtosecond pulsed excitation using Equation (1):

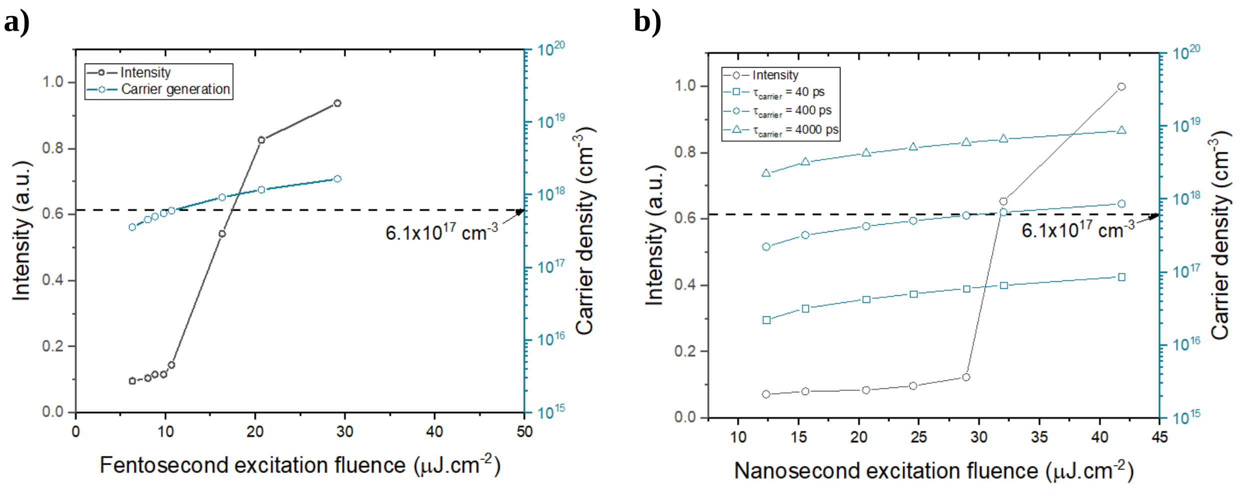

Figure S15. Integrated power-dependent PL and carrier density as a function of excitation fluence for a) femtosecond laser and b) nanosecond laser excitation.

The carrier density at optical gain threshold using Equation 1 (assuming the carrier recombination rate is negligible compared with the carrier generation rate), as shown in Figure S15, is around 6.1x10^{17} cm^{-3}, similar to that of perovskite lasers (approximately 10^{18} cm^{-3} carriers[15]).

For pulse excitation comparable with the carrier lifetime ($\tau_{pulse} \sim \tau_{carrier}$), the carrier density should be derived by considering both the carrier injection and carrier recombination simultaneously. The longer pulse can be regarded as quasi-continuous-wave excitation due to similar dynamics as in continuous-wave excitation[15]. Equilibrium is realized when G is equal to the carrier recombination rate, therefore (Equation 2):

$$G = \frac{n}{\tau_{carrier}} \, (=) \, n = \tau_{carrier} \times (1 - R)(1 - e^{-\alpha d}) \frac{P_{ns}}{d \frac{hc}{\lambda}}$$

Where P_{ns} is peak power density. The carrier lifetime is obtained by:

$$\tau_{carrier}{}^{-1} = \tau_a{}^{-1} + \tau_b{}^{-1} + \tau_c{}^{-1}$$

Where a, b and c correspond to the first-order, second-order and third-order recombination rate coefficients. The first-order term contains radiative recombination – we extract it from PL lifetime at low carrier densities (Figure 3c, approximately 20 ns, in good agreement with previous reports[9]). The second-order term contains free electron-hole recombination. The third-order term contains Auger recombination. The total carrier lifetime is no longer than any of the three individual lifetimes. At the high fluences required for perovskite lasing ($\sim 10^{18}$ cm^{-3}) [15], the carrier lifetime is dominated by Auger lifetime ($\tau_{carrier} \sim \tau_c$). Figure S15b compares the carrier density for a carrier lifetime of 40 ps, 400 ps and 4000 ps.

As shown in Figure S15b, the carrier density at threshold with femtosecond laser excitation (6.1×10^{17} cm^{-3}) is matched by the carrier density at threshold with nanosecond laser excitation for a Auger lifetime of 100-400 ps.

The analysis presented here does not substitute direct measurement of Auger lifetime using transient absorption and photoluminescent techniques. However, as noted in the main text, due to focusing and signal limitations, we could not characterize Auger recombination using transient absorption. We could not probe a single superlattice directly; rather, we were limited to probing a larger area of the substrate. Thus, any signal obtained from the SLs overlapped with a stronger signal from the surrounding uncoupled QDs.

References

1. J. Maes, L. Balcaen, E. Drijvers, Q. Zhao, J. De Roo, A. Vantomme, F. Vanhaecke, P. Geiregat, Z. Hens, *The Journal of Physical Chemistry Letters* **2018**, *9*, 3093.

2. V. Blum, R. Gehrke, F. Hanke, P. Havu, V. Havu, X. Ren, K. Reuter, M. Scheffler, *Computer Physics Communications* **2009**, *180*, 2175.

3. V. Havu, V. Blum, P. Havu, M. Scheffler, *Journal of Computational Physics* **2009**, *228*, 8367.

4. X. Ren, P. Rinke, V. Blum, J. Wieferink, A. Tkatchenko, A. Sanfilippo, K. Reuter, M. Scheffler, *New Journal of Physics* **2012**, *14*, 053020.

5. M. Yuan, L. N. Quan, R. Comin, G. Walters, R. Sabatini, O. Voznyy, S. Hoogland, Y. Zhao, E. M. Beauregard, P. Kanjanaboos, Z. Lu, D. H. Kim, E. H. Sargent, *Nature Nanotechnology* **2016**, *11*, 872.

6. Y. Jiang, M. Cui, S. Li, C. Sun, Y. Huang, J. Wei, L. Zhang, M. Lv, C. Qin, Y. Liu, M. Yuan, *Nature Communications* **2021**, *12*, DOI 10.1038/s41467-020-20555-9.

7. J. A. McGuire, J. Joo, J. M. Pietryga, R. D. Schaller, V. I. Klimov, *Accounts of Chemical Research* **2008**, *41*, 1810.

8. F. Wang, Y. Wu, M. S. Hybertsen, T. F. Heinz, *Physical Review B* **2006**, *73*, DOI 10.1103/physrevb.73.245424.

9. Q. Li, C. Li, Q. Shang, L. Zhao, S. Zhang, Y. Gao, X. Liu, X. Wang, Q. Zhang, *The Journal of Chemical Physics* **2019**, *151*, 211101.

10. N. S. Makarov, S. Guo, O. Isaienko, W. Liu, I. Robel, V. I. Klimov, *Nano Letters* **2016**, *16*, 2349.

11. C. R. Kagan, E. Lifshitz, E. H. Sargent, D. V. Talapin, *Science* **2016**, *353*, DOI 10.1126/science.aac5523.

12. T. Wu, J. Li, Y. Zou, H. Xu, K. Wen, S. Wan, S. Bai, T. Song, J. A. McLeod, S. Duhm, F. Gao, B. Sun, *Angewandte Chemie International Edition* **2020**, *59*, 4099.

13. Y. Dong, Y.-K. Wang, F. Yuan, A. Johnston, Y. Liu, D. Ma, M.-J. Choi, B. Chen, M. Chekini, S.-W. Baek, L. K. Sagar, J. Fan, Y. Hou, M. Wu, S. Lee, B. Sun, S. Hoogland, R. Quintero-Bermudez, H. Ebe, P. Todorovic, F. Dinic, P. Li, H. T. Kung, M. I. Saidaminov, E. Kumacheva, E. Spiecker, L.-S. Liao, O. Voznyy, Z.-H. Lu, E. H. Sargent, *Nature Nanotechnology* **2020**, *15*, 668.

14. Y. K. Wang, F. Yuan, Y. Dong, J. Y. Li, A. Johnston, B. Chen, M. I. Saidaminov, C. Zhou, X. Zheng, Y. Hou, K. Bertens, H. Ebe, D. Ma, Z. Deng, S. Yuan, R. Chen, L. K. Sagar, J. Liu, J. Fan, P. Li, X. Li, Y. Gao, M. K. Fung, Z. H. Lu, O. M. Bakr, L. S. Liao, E. H. Sargent, *Angewandte Chemie International Edition* **2021**, DOI 10.1002/anie.202104812.

15. J. Qin, X.-K. Liu, C. Yin, F. Gao, *Trends in Chemistry* **2021**, *3*, 34.
